# Decreasing single-use plastic within the pneumatic tube system during medication transport

**DOI:** 10.1017/ash.2026.10385

**Published:** 2026-05-12

**Authors:** Shreya M. Doshi, Demilade Haastrup, Xiaoyan Song

**Affiliations:** 1 Division of Infectious Diseases, Children’s National Hospital, Washington, DC 20010, USA; 2 Department of Pediatrics, George Washington University, Washington, DC 20010, USA; 3 Division of Pharmacy, Children’s National Hospital, Washington, DC 20010, USA

Dear Editor,

Pneumatic tube systems (PTS) have become a common mechanism for transporting items such as medications, documents, supplies, and lab specimens within large healthcare facilities. Contamination of PTS has been linked to a pseudo-outbreak in a large hospital.^1^ To mitigate the risk of cross-contamination, our pharmacy initially adopted the universal use of plastic bags as secondary containment. In 2024, this practice prompted a collaboration between Pharmacy and Infection Prevention and Control, which subsequently led to a reduction in plastic bag use while maintaining the safety of the PTS. Our initiative illustrates how clearly defined policy changes developed with Infection Prevention can deliver measurable safety, cost, and environmental benefits. The UK Sustainable Quality Improvement^2^ approach calls for QI initiatives to consider the triple bottom line of patient safety and experience, environmental impact, and financial value, integrating sustainability into routine quality work rather than treating it as a separate aim, as demonstrated by this project.

About the policy: In 2024, our Pharmacy and Infection Prevention and Control team re-evaluated routine bagging policy within the pneumatic tube carriers. The revised policy (Table 1) specifies when carrier-only transport is allowed and when bagging remains necessary. Carrier-only transport (without a secondary plastic bag) is appropriate when the item is clean; outer packaging is intact, and the item is not a return item. Items must meet the pneumatic tube limits of 2.2 pounds or 1 liter, and carrier must close and latch fully with no protruding paperwork or packaging. For medications, the pharmacist’s final verification must be complete before dispatching. Bulk medications in sealed manufacturer or pharmacy packaging and properly capped oral medications do not require plastic bagging. Bulk medications include sealed multidose bottles, blister cards, inhalers, ointments, and creams when the exterior packaging is intact. Properly capped oral medications include pharmacy-dispensed liquid preparations in bottles or oral syringes with secure caps that have been checked for tight closure and an intact seal. These items qualify for carrier-only transport provided they are not on the do-not-tube list, do not exceed weight or volume limits, and show no signs of leakage risk.

**Table 1.** Pneumatic tube transport and bagging criteria used to reduce single-use plastic

Decision point	Carrier-only transport (no bag)	Bag required	Do not tube
**Item type**	Clean medications/supplies with intact outer packaging (excluding returns)	Laboratory specimens; return medications; selected hazardous materials (USP <800>)	Items labeled “Do Not Tube” or prohibited by institutional policy
**Packaging condition**	Intact packaging; no leakage risk	When needed to prevent leakage or protect seals (user discretion)	Leaking, poorly sealed, or damaged items
**System limits**	≤2.2 lb. and ≤1 L; carrier fully closes/latches; nothing protrudes	Same limits apply	Exceeds weight/volume limits or cannot be secured
**Pharmacy safeguard**	Pharmacist final verification completed before dispatch	Pharmacist final verification completed before dispatch	Pharmacist final verification completed before dispatch
**High-risk categories**	—	Hazardous medications permitted for tubing must be double-bagged per policy	Examples include blood/protein products, investigational drugs, controlled substances requiring signatures, flammable/pressurized items, TPN/lipids, >1 L IV fluids, large/heavy items, fragile glass vials (per policy)

Cleaning, decontamination, and event reporting procedures were the safety backstop for suspected contamination or spills.

A single-use plastic bag remains mandatory for laboratory specimens, for return medications, and selected hazardous medications per USP 800 guidelines and as identified in policy. Where institutional policy allows any hazardous medication to travel by pneumatic tube, it must be double bagged, with both bags sealed and compatible with the facility’s decontamination procedures. If local policy prohibits pneumatic transport of a given hazardous agent, that item must not be tubed. These rules apply in addition to existing exclusions for products sensitive to agitation, blood or protein products, high-value or fragile preparations, controlled or investigational drugs that require signatures or chain of custody, flammable or pressurized items, total parenteral nutrition and lipid emulsions, intravenous solutions larger than 1 liter, large or heavy items, and glass vials with a meaningful risk of breakage. Established shutdown, decontamination, and reporting procedures served as the safety backstop enabling plastic reduction, with Facilities and Infection Prevention and Control leading responses to suspected contamination or spills, including system cleanout and event reporting as indicated.

Using these guardrails, a joint Pharmacy, Infectious Diseases, and Sustainability effort reframed “no bag needed” scenarios and updated workflows so that single-use plastic bags are used only when required. Nursing staff expressed strong support for the initiative. We aimed to reduce pharmacy plastic bags from more than 2 million per year to fewer than 300,000 and to sustain that level. This QI achieved a 60% reduction in bag use, over $30,000 in annual procurement savings, and improved tube turnaround time, with policy re-evaluated at six months. These financial savings are significant and helped fund additional pharmacy technician and medication delivery support. The primary limitation of this project was adherence to policy. After observing continued occasional double-bagging practice, we implemented ongoing educational initiatives for pharmacy staff to reinforce adherence.

Reducing avoidable plastic aligns with the one-health mission. Additionally, mitigating waste provides a sense of purpose to employees and has been reported to enhance employee retention. U.S. hospitals generate an estimated 14,000 tons of waste daily, much of it single-use, and experts recommend culture and policy change as a core strategy.^3^ A Canadian NICU QI initiative achieved plastic reductions using a different approach.^4^ Streamlining plastic packaging is often low risk with respect to safety, effectiveness, and affordability, and our experience shows how to do this in practice.^5^ The Lancet Countdown on health and plastics describes plastics as a growing danger and estimates health-related economic losses that exceed 1.5 trillion U.S. dollars each year.^6^ Human studies now report micro and nano plastics in carotid atheromas, with the presence of these particles associated with a higher risk of myocardial infarction, stroke, or death.^7^ Planetary health considerations should be embedded across pharmaceutical supply chains, and Infection Prevention and Control experts have a central role in doing so safely.^8,9^
